# Evaluating the Flipped Classroom Approach for Enhancing Orthodontic and Cephalometric Competencies in Dental Undergraduates: A Quasi-experimental, Non-randomized Study

**DOI:** 10.7759/cureus.108995

**Published:** 2026-05-16

**Authors:** Hitesh R Sawant, Parag Gangurde, Amit Patil, Sheetal M Jadhav, Swati Kale, Harshita R Sheth

**Affiliations:** 1 Orthodontics and Dentofacial Orthopaedics, Bharati Vidyapeeth (Deemed to be University) Dental College and Hospital, Navi Mumbai, IND; 2 Conservative Dentistry and Endodontics, Bharati Vidyapeeth (Deemed to be University) Dental College and Hospital, Navi Mumbai, IND; 3 Prosthodontics, Bharati Vidyapeeth (Deemed to be University) Dental College and Hospital, Navi Mumbai, IND; 4 Pediatric and Preventive Dentistry, Bharati Vidyapeeth (Deemed to be University) Dental College and Hospital, Navi Mumbai, IND

**Keywords:** active learning, cephalometric analysis, dental education, flipped classroom, student engagement

## Abstract

Background and objective

Cephalometric analysis is a critical component of orthodontic diagnosis, requiring precise measurements, interpretation, and practical application. Traditional lecture-based instruction may not fully engage students or foster the analytical and problem-solving skills necessary for clinical practice. The flipped classroom model, which integrates pre-class learning with interactive in-class activities, provides a potentially more effective approach. This study aimed to evaluate the effectiveness of the flipped classroom model versus traditional lectures in improving knowledge, engagement, and satisfaction among undergraduate dental students studying cephalometric analysis.

Methods

A quasi-experimental, non-randomized, two-group pre-test/post-test design was used, involving 72 final-year undergraduate dental students. Participants were evenly assigned to a control group (traditional lecture) and an experimental group (flipped classroom). The flipped classroom group received pre-class instructional videos, guided reading materials, and a self-assessment quiz, followed by an interactive session featuring case-based discussions, hands-on tracing, and peer teaching. The control group received a conventional lecture covering the same content. Outcomes measured included knowledge gain (assessed using multiple-choice questions (MCQs) and clinical scenario questions) and engagement/satisfaction (assessed using a Likert-scale questionnaire). Statistical analysis was conducted using paired and independent t-tests, two-way ANOVA, and effect size calculations.

Results

The flipped classroom group achieved significantly higher post-test scores (mean = 7.69, standard deviation (SD) = 0.67) compared to the lecture group (mean = 5.28, SD = 1.21), t(70) = 10.49, p < 0.001, Cohen’s d = 2.47. No significant gender differences were found, and no interaction between model and gender was observed. Engagement and satisfaction scores were markedly higher in the flipped group.

Conclusions

The flipped classroom model led to significant improvements in cognitive outcomes, knowledge retention, engagement, and student satisfaction compared to traditional lectures. Its learner-centered, active approach is particularly well-suited for complex orthodontic topics and has the potential for broader implementation in dental education.

## Introduction

The success of any orthodontic treatment depends on an accurate diagnosis and careful treatment planning, both of which require high precision from the practitioner [1]. Topics such as cephalometric analysis, a cornerstone of orthodontic diagnosis, entail precise measurements and interpretation of cephalometric radiographs to assess craniofacial skeletal and dental relationships [2]. The conventional method of teaching cephalometric analysis, typically involving lectures and textbook readings, may not fully engage students or adequately develop their analytical and problem-solving skills, which are essential for interpreting cephalometric radiographs and devising treatment plans.

The flipped classroom model offers several advantages over traditional teaching methods. By providing instructional content before class, students can learn at their own pace, rewatching videos or revisiting online modules as needed to master complex concepts. This approach allows for more personalized learning experiences and accommodates diverse learning styles. Additionally, when students arrive in class having already been exposed to the core content, they are better prepared to engage in active learning exercises, resulting in more meaningful interactions with both peers and instructors [3].

The inverted classroom model reconfigures the learning environment by positioning students as active participants in their education. In this approach, students first engage with instructional content through pre-class materials such as videos, readings, or interactive modules. Class time is then used for discussions, problem-solving, and collaborative activities, enabling students to apply their pre-acquired knowledge in practical contexts. This inversion of the traditional lecture format has been shown to enhance student involvement and promote critical thinking [4]. Within the domain of cephalometric analysis, a fundamental component of orthodontic and maxillofacial studies, this active engagement is particularly important, as students must understand complex spatial relationships and essential analytical techniques for accurate diagnosis and treatment planning. Therefore, this study aims to evaluate the effectiveness of the flipped classroom model among undergraduate dental students by comparing the academic performance of students taught using the flipped classroom approach versus traditional lectures on cephalometric analysis. The objectives are to assess student engagement and satisfaction and to evaluate knowledge retention over time.

## Materials and methods

This was a quasi-experimental, non-randomized, two-group pre-test/post-test study conducted in accordance with the principles of the Declaration of Helsinki and approved by the Institutional Ethical Review Board. The research was carried out in the Department of Orthodontics and Dentofacial Orthopedics at a reputable dental college to evaluate the effectiveness of the flipped classroom model in enhancing undergraduate dental students’ cognitive understanding of cephalometric analysis compared to traditional didactic teaching.

Recruitment and allocation

A total of 72 final-year undergraduate dental students enrolled in the Bachelor of Dental Surgery program were recruited for this study. Cephalometric analysis was selected as the topic for the teaching intervention. The students were randomly assigned to two equal groups: a control group (n = 36) and an experimental group (flipped classroom, n = 36). Eligible participants had not received any prior formal instruction or training in cephalometric analysis and provided voluntary consent to participate.

Sample size

The sample size for this study was calculated using the formula for comparing the means of two independent groups:

$n=(&mu;1​&minus;&mu;2​)22(Z&alpha;/2​+Z&beta;​)2\sigma2​​$

Where n = required sample size per group; Zα/2​ = standard normal deviate corresponding to the desired level of significance (1.96 for 95% confidence interval (CI); Zβ​ = standard normal deviate corresponding to study power (0.84 for 80% power); σ = estimated pooled standard deviation (SD); and μ1​−μ2​ = expected difference in mean post-test scores between groups.

Based on findings from previous educational intervention studies and a pilot estimation, a moderate-to-large effect size was expected. Using a significance level of 5% (α = 0.05) and a study power of 80% (β = 0.20), the minimum required sample size was calculated as 36 participants per group. Therefore, a total of 72 final-year undergraduate dental students were included, with 36 students allocated to the traditional lecture group and 36 to the flipped classroom group. This sample size was considered sufficient to detect a statistically significant difference in learning outcomes between the two instructional methods.

Inclusion criteria

Final-year undergraduate dentistry students enrolled in the orthodontics course during the study period were included. Students were considered eligible if they had not previously received formal training in cephalometric analysis. Only individuals who attended both the pre-test and post-test assessment sessions and provided informed consent were eligible to participate.

Exclusion criteria

Students who had received additional training or clinical experience in cephalometric analysis beyond the standard undergraduate program were excluded. Additionally, those who did not attend the intervention sessions or evaluation procedures were not considered. Students who declined to participate, withdrew consent during the study, or failed to complete the pre-class learning activities or post-intervention assessments were also excluded from the final analysis.

Lesson plan

Students in the control group received a conventional 90-minute lecture delivered by an experienced faculty member from the department. The lecture covered the following core topics: (I) identification and description of cephalometric landmarks, (II) basic and advanced cephalometric tracing techniques, and (III) interpretation of cephalograms using Steiner’s analysis. The session was conducted in a standard classroom setting using audiovisual aids, including PowerPoint presentations and annotated diagrams. No additional pre-class or post-class materials were provided to this group.

The flipped classroom group underwent a blended instructional approach combining pre-class preparation with in-class interactive activities. One week before the scheduled session, students in the experimental group were provided access to online learning materials, including three short pre-recorded instructional videos of approximately 10 minutes each. The videos covered (I) cephalometric landmarks and their significance, (II) step-by-step cephalometric tracing techniques, and (III) principles and application of Steiner’s analysis. In addition, students received a structured, guided reading handout summarizing the key concepts and a self-assessment quiz based on the video and reading content to reinforce understanding and identify knowledge gaps.

Following the completion of the pre-class activities, students participated in a 90-minute face-to-face interactive session facilitated by faculty. The session included (I) small group discussions of clinical case scenarios, emphasizing the interpretation of cephalograms, (II) hands-on tracing exercises on cephalometric radiographs using acetate sheets and light boxes, supervised by faculty, and (III) peer teaching sessions where students collaboratively discussed findings and clarified doubts with faculty support. This structure was designed to promote active learning, critical thinking, and peer collaboration in a clinically relevant context.

Outcome assessment

To evaluate the effectiveness of the instructional interventions, both groups completed an identical set of assessments administered before and after the teaching sessions. The assessment tool consisted of (I) five multiple-choice questions (MCQs) designed to evaluate factual knowledge and application of cephalometric principles and (II) five short-answer clinical scenario-based questions aimed at testing higher-order cognitive skills, including interpretation and decision-making.

The same set of questions was used to assess the overall performance of the students. All assessments were administered in a supervised classroom environment and collected immediately after completion. Scoring was done by 10 examiners within the department with above 10 years of experience. All collected data, including test scores and survey responses, were entered into Microsoft Excel (Microsoft Corporation, Redmond, WA) and exported to IBM SPSS Statistics (IBM Corp., Armonk, NY) for statistical analysis. Data were double-checked for entry errors before analysis. Descriptive statistics were calculated for all outcome variables. Mean and SD were computed for pre-test and post-test scores within each group (control and experimental). Frequencies and percentages were calculated for categorical data, including survey responses.

Two primary outcome variables were analyzed: (I) knowledge gain - defined as the difference between post-test and pre-test scores (within-group comparison was assessed using a paired sample t-test to evaluate cognitive improvement in both the control and experimental groups individually; between-group comparison was assessed using an independent sample t-test and ANOVA to compare the knowledge gain (post-test minus pre-test scores) between the two groups; and (II) engagement and satisfaction - measured using a validated Likert-scale-based questionnaire.

Normality of continuous data was assessed using the Shapiro-Wilk test and Q-Q plots. Homogeneity of variances was verified using Levene’s test before applying *t*-tests and ANOVA. In cases where assumptions of normality or homogeneity were violated, non-parametric alternatives (e.g., Wilcoxon signed-rank test or Mann-Whitney U test) were employed. Effect sizes (Cohen’s d) were calculated for all t-tests to quantify the magnitude of observed differences. Partial eta-squared was reported for ANOVA analyses to indicate the proportion of variance explained by the intervention. A two-tailed p-value < 0.05 was considered statistically significant for all comparisons. Bonferroni corrections were applied in cases of multiple comparisons to control for type I error.

## Results

**Figure 1:**
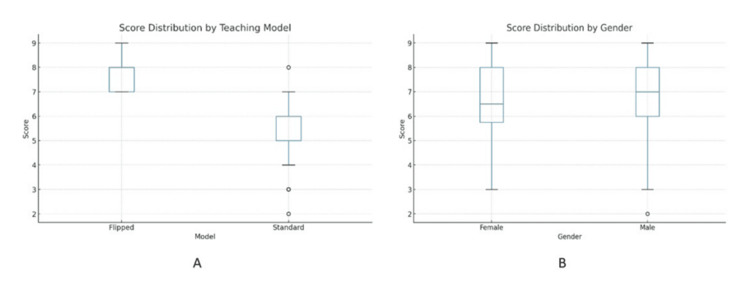
(A) Score distribution by teaching model and (B) gender Credit: Dr. Hitesh Sawant

**Figure 2:**
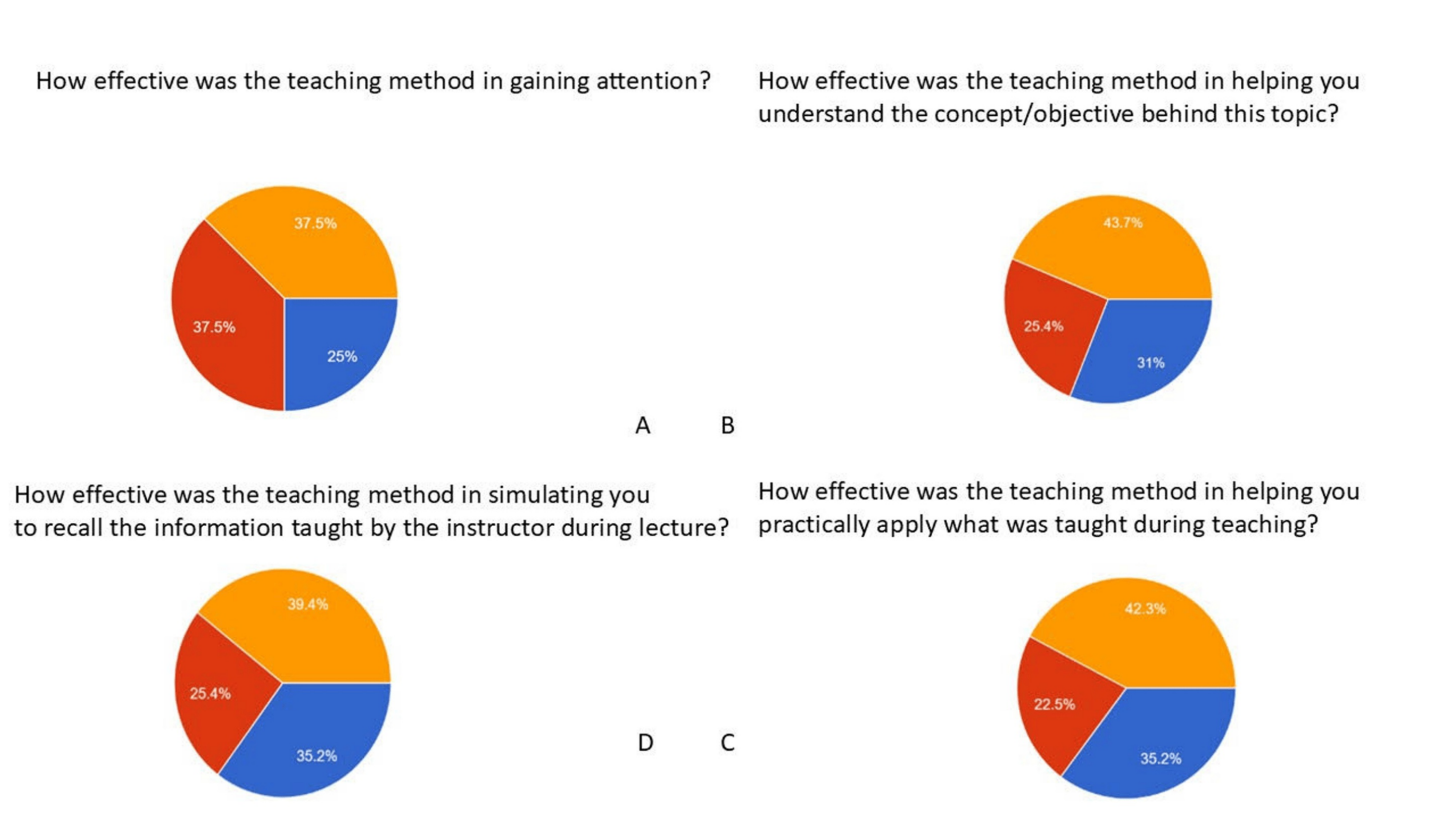
Score distribution for (A) attention, (B) understanding, (C) recall, and (D) effectiveness Credit: Dr. Hitesh Sawant

An independent samples t-test was conducted to compare scores between the flipped classroom model and the standard lecture model. Scores were significantly higher for the flipped classroom (mean = 7.69, SD = 0.67) than for the standard lecture (mean = 5.28, SD = 1.21), t(70) = 10.49, p < 0.001, Cohen’s d = 2.47, indicating a large effect size. An independent samples t-test comparing male (mean = 6.42, SD = 1.66) and female (mean = 6.56, SD = 1.46) students found no statistically significant difference, t(70) = -0.38, p = 0.708, Cohen’s d = 0.09 (Figures 1, 2).

A two-way ANOVA was conducted to examine the effects of teaching model (flipped vs. standard) and gender (male vs. female) on scores. The main effect of the teaching model was statistically significant (F(1, 68) = 110.18, p < 0.001, partial η² = 0.618), with the Flipped classroom yielding higher scores. The main effect of gender was not significant (F(1, 68) = 1.40, p = 0.241, partial η² = 0.020). The interaction between model and gender was also not significant (F(1, 68) = 0.016, p = 0.900, partial η² < 0.001) (Table 1).

**Table 1:** Results of the analysis

Comparison	Groups	Mean	Standard deviation	Test used	Test statistic	p-value	Effect Ssze	Interpretation
Teaching model comparison	Flipped classroom	7.69	0.67	Independent t-test	t(70) = 10.49	< 0.001	Cohen’s d = 2.47	Significantly higher scores in the flipped classroom group
Teaching model comparison	Standard lecture	5.28	1.21	Independent t-test	—	—	—	—
Gender comparison	Male	6.42	1.66	Independent t-test	t(70) = -0.38	0.708	Cohen’s d = 0.09	No significant difference between genders
Gender comparison	Female	6.56	1.46	Independent t-test	—	—	—	—
Two-way ANOVA (main effect: teaching model)	Flipped vs. standard	—	—	Two-way ANOVA	F(1,68) = 110.18	< 0.001	Partial η² = 0.618	Significant effect of teaching model
Two-way ANOVA (main effect: gender)	Male vs. female	—	—	Two-way ANOVA	F(1,68) = 1.40	0.241	Partial η² = 0.020	No significant gender effect
Interaction effect (model × gender)	—	—	—	Two-way ANOVA	F(1,68) = 0.016	0.9	Partial η² < 0.001	No interaction effect

## Discussion

As dental education evolves in response to advances in pedagogical research, the implementation of various instructional strategies has become increasingly crucial for improving the educational experience for dental undergraduate students. Among these strategies, the inverted classroom model has gained prominence, particularly in subjects that require high levels of involvement and practical application from students, such as cephalometric analysis. This model contrasts severely with traditional lecture-based approaches, where knowledge dissemination occurs mainly through passive listening. Traditional lectures have dominated dental education for decades; however, they often do not meet the diverse learning styles of contemporary students and tend to limit interactivity, which is fundamental to complex subjects.

A substantial body of literature corroborates these findings. Among them is a study conducted among Jordanian dental students exposed to a blended learning strategy that included a flipped classroom component. These students not only scored higher in assessments but also showed improved participation in digital learning forums [5]. This level of engagement likely fostered deeper learning and reinforced the correlation between active participation and academic performance.

Flipped classroom models have been shown to enhance cognitive learning outcomes when coupled with collaborative and active learning strategies [6]. Research indicates that interactive teaching methods, such as those employed in the inverted classroom model, facilitate deeper knowledge acquisition, as students can be directly involved with their colleagues and instructors in higher-order thought exercises during class sessions. This active participation is essential in areas such as cephalometric analysis, where conceptual understanding and analytical skills are fundamental. Learning environments characterized by active learning strategies not only improve cognitive involvement but also significantly contribute to students’ intrinsic motivation [7]. The collaborative nature of the inverted classroom provides immediate feedback and effective clarification, which is particularly important in a technically demanding field like dentistry.

The benefits of the flipped model are especially significant in the context of dental education, where clinical reasoning, psychomotor skills, and the integration of theoretical knowledge into practice are critical. Video-driven pre-class learning in dental education has been found to enhance not only theoretical knowledge but also practical performance and higher-order thinking skills. However, as researchers note, the success of such models depends heavily on the quality and structure of pre-class materials, emphasizing the need for well-designed instructional videos and guided self-assessment tools [8]. Beyond improved academic performance, the flipped classroom approach fosters inclusivity and narrows the performance gap between high- and low-achieving students. Peer interaction and collaboration encourage underperforming students to engage more actively, thereby enhancing their learning outcomes [9]. Our findings are consistent with this, as increased peer interaction during tracing sessions helped solidify anatomical concepts across all ability levels.

In addition, the integration of technology into the inverted classroom design contributes significantly to improved learning outcomes. The use of digital tools allows a diverse range of learning experiences, meeting various learning preferences [10]. For example, video lectures can be paused, rewound, and revisited, allowing students to master challenging concepts at their own pace. They also provide dental students with a visual and kinesthetic learning experience that traditional lectures cannot offer. These tools are particularly valuable for teaching complex procedures and anatomical structures, such as those involved in cephalometric analysis [11]. When students visualize the anatomical landmarks and practice tracing techniques repeatedly with digital aids before class, they arrive better prepared for hands-on, faculty-guided exercises. Synchronous and asynchronous interactions facilitated by digital platforms further enhance accessibility and flexibility, which are crucial to accommodating the demands of dental education.

Moreover, the flipped model supports the development of self-directed learning, a key competency in dental education. It helps students take ownership of their learning, allowing them to revisit difficult concepts at their own pace and improving long-term retention [12]. Additionally, the flipped classroom environment promotes soft skills that are often underemphasized in traditional teaching. Active participation in class discussions, peer collaborations, and problem-solving tasks enhances communication, teamwork, and leadership skills, all of which are vital for successful dental practice [13]. Instructors also benefit from this model, as it allows more time during class to identify struggling students, offer personalized support, and focus on clinical application. Another emerging advantage is the model's adaptability to hybrid or fully online formats, which is particularly useful during disruptions such as the COVID-19 pandemic. Several studies have confirmed that the flipped classroom can be seamlessly integrated into remote learning systems while maintaining, or even enhancing, educational outcomes [14,15].

A growing body of literature illustrates that these passive learning environments may not stimulate the same level of cognitive commitment as active learning methodologies, such as those employed in flipped classrooms [16]. Students in traditional environments often report feelings of disconnection during lectures, as the lack of interactive elements can lead to decreased information retention. This passive absorption of knowledge is particularly detrimental in fields that rely heavily on critical thinking and problem-solving skills, such as dental practice. In addition, students' preferences regarding teaching methods cannot be overlooked when evaluating the effectiveness of traditional lectures. Studies conducted with large student populations show a preference for learning environments that encourage active participation rather than passive memorization.

This preference highlights an emerging change in educational expectations, where students seek learning experiences that promote deeper understanding and practical application of knowledge. The inability of traditional lectures to adapt to these preferences can lead to decreased student satisfaction, potentially affecting their overall educational experience. In addition, the limitations of traditional lectures are particularly pronounced when considering the practical application of cephalometric analysis within the dental field. Mastery of cephalometric concepts is not only about content delivery but also requires students to engage in spatial reasoning, critically analyze data, and perform practical analyses. Traditional lectures often do not incorporate these elements, leaving a gap in practical training that can impede students’ ability to translate theoretical knowledge into clinical practice.

In summary, while traditional lectures serve important functions in the dissemination of information and fundamental learning, their effectiveness in promoting engagement, knowledge retention, and student satisfaction appears limited compared to more interactive educational formats. Recognizing these limitations is crucial for educators who seek to optimize learning outcomes in dental education, particularly in specialized areas such as cephalometric analysis. Ongoing investigation into students’ experiences and preferences will be vital for shaping pedagogical approaches that meet both educational objectives and the needs of students.

In light of these findings and those of the present study, it is evident that the flipped classroom is not merely a temporary innovation but a sustainable and superior pedagogical strategy for dental education. When implemented effectively, it can enhance academic performance, foster critical thinking, support personalized and self-paced learning, and better prepare students for clinical practice.

## Conclusions

Through self-paced pre-class engagement and active in-class application, students demonstrated improved comprehension and retention of cephalometric concepts, suggesting that the flipped classroom approach offers a significant advantage over traditional lecture-based teaching in dental education. By encouraging deeper understanding, this interactive approach helped students transition from passive listening to active engagement. Higher levels of involvement and satisfaction, promoted by a dynamic, cooperative learning environment, coincided with enhanced academic performance. Notably, these benefits were observed across all genders, demonstrating the approach's versatility and inclusivity. Overall, these findings support the integration of flipped classroom methodologies into the dental curriculum, particularly for challenging disciplines such as orthodontics and cephalometry, to improve learning outcomes and enrich the overall educational experience.
